# Stewardship and social justice: implications of using the precautionary principle to justify burdensome antimicrobial stewardship measures

**DOI:** 10.1007/s40592-024-00224-z

**Published:** 2024-12-04

**Authors:** Tess Johnson

**Affiliations:** 1 Ethox Centre, Nuffield Department of Population Health, University of Oxford, Oxford, UK; 2 Pandemic Sciences Institute, Nuffield Department of Medicine, University of Oxford, Oxford, UK

**Keywords:** Public health ethics, Precautionary principle, Justice, Antimicrobial resistance, Stewardship

## Abstract

Antimicrobial resistance has been termed a ‘silent pandemic’, a ‘hidden killer.’ This language might indicate a threat of significant future harm to humans, animals, and the environment from resistant microbes. If that harm is uncertain but serious, the precautionary principle might apply to the issue, and might require taking ‘precautionary measures’ to avert the threat of antimicrobial resistance, including stewardship interventions like antibiotic prescription caps, bans on certain uses in farming sectors, and eliminating over-the-counter uses of antibiotics. The precautionary principle is a useful tool in ethical analyses of antimicrobial stewardship measures, but as I argue in this article, it ought not be used as a standalone tool. The principle considers the magnitude of harms to be averted and those arising from precautionary measures, but—importantly—it does not consider the distribution of those harms. That may raise issues of social justice if the harms of stewardship measures befall already disadvantaged populations. To avoid this blind spot in ethical analysis using the precautionary principle, it ought never be used alone, but rather always alongside justice-considering ethical concepts such as reciprocity, benefit-sharing, or a just transition.

## Introduction

Antimicrobial resistance (AMR) occurs when microbes—bacteria, viruses, fungi, and parasites—evolve to resist the environmental substances or drugs that would normally kill them or stop them replicating. This has serious implications for the treatment of diseases, and even routine surgeries. AMR has variously been termed a ‘hidden killer’ (Rosencranz 2022), a ‘silent pandemic’ (Cullen 2023), and an ‘urgent threat to life as we know it’ (UN Environment Programme 2023). Tensions arise between individual and collective interests when it comes to addressing AMR, and there are burdens associated both with intervening via stewardship, and with not intervening (Pokharel et al. 2024). With projections of up to 10 million deaths per year and an economic cost of 100 trillion US dollars resulting from AMR by 2050 (O’Neill 2014), attention-grabbing language may not appear out of place when discussing the threat of AMR.

Given that AMR does pose a threat of serious harm to humans (not to mention animals and the environment), the issue might be appropriately considered using a particular ethical concept that aims to guide policy action in the face of uncertainty and potential catastrophe: the precautionary principle (PP) (Munthe 2020). If the harms of AMR are expected to be serious enough, even if we are uncertain about the mechanisms by which they might occur or other details, then PP would still apply.^1^ In general, the principle^2^ holds that precautionary measures to avert a threat posing serious harm ought to be taken, even under conditions of uncertainty. It involves assessing measures that might avert the harm, and asserting that an authority can be held responsible for taking action. Let us assume, for now, that AMR fulfils the requirements for appropriate application of PP. In that case, it might be argued that precautionary measures ought to be taken that could include stewardship efforts aiming to optimise antibiotic use in humans, animals, and the environment, to reduce unnecessary contributions to the problem of resistance and increase access in settings where it’s inadequate. Stewardship might include bans on uses of human-critical antibiotics in livestock feed, capping doctors’ antibiotic prescription numbers, or eliminating the supply-chains for over-the-counter (without prescription) sales of antibiotics. Insofar as PP provides a justification for these precautionary measures, particularly without the need for absolute certainty that 10 million people will die per year by 2050 from AMR, it seems a useful ethical concept for guiding action on uncertain but potentially catastrophic threats.

Yet, there is a problem with policy that is guided by PP when it’s used as a stand-alone ethical principle: PP considers the magnitude of AMR-related harms and even the proportionality of harms that might arise from precautionary measures such as stewardship, but *not* the justice implications of those stewardship measures, including how their harms are distributed among individuals and populations and how these people are treated. This leads to the problem that is the subject of this special issue, and the rest of this editorial: AMR and social justice. As I argue here, and as other papers in this special issue highlight, there is a potential tension between the minimisation of harms and promotion of  social justice, because failures of social justice may be caused or perpetuated by measures designed to address AMR.

This may be particularly the case for coercive or burdensome antimicrobial stewardship measures: insofar as the potential harms of those measures are greater than non-coercive or less burdensome measures, they are particularly in need of ethical analysis. Unfair distribution of harms or treating people unfairly is less an issue of justice if those harms are insignificant, whereas it is more ethically important that larger harms are distributed fairly. Consider two burdensome policies. First, in efforts to avert resistance, policymakers may ban farmers' use of human-critical antibiotics in medicated feed for livestock to prevent herd-level infection. This may impose burdens on a particular (perhaps already disadvantaged) population that is reliant on frequent use of antibiotics—in this context, perhaps farmers in a low-income country who are raising animals on razor-thin profit margins with little state support. Such measures have already been proposed, and critiqued for their social justice implications (Just Transitions Working Group 2023; Reid 2020). Second, consider another burdensome stewardship measure, this one targeting communities who are reliant on access to antibiotics without a prescription, often dispensed through community pharmacies. Banning over-the-counter access to antibiotics in this way may disproportionately impact those who have less access to prescribers, and to healthcare more broadly. In this paper, I discuss how ethical analyses of proposed stewardship measures have at times relied on principles like PP without reference to justice-related concepts alongside PP. I argue that this has allowed for the perpetuation of failures of social justice due to PP not incorporating considerations of the distribution of harms from precautionary measures alongside considerations of their comparative magnitude against harms averted. To make my argument, I first discuss examples of burdensome antimicrobial stewardship policies justified by reference to PP, before going on to explain how this relates to problems of social justice. To cap off my argument, I present a possible solution: the supplementation of ethical concepts and approaches for policymaking like PP with justice-related concepts. Finally, I outline the contributions of other papers in this special issue.

## Antimicrobial resistance and the precautionary principle

PP traces its origins to environmental ethics. It was developed in relation to reversing the burden of proof and reducing the need for extensive evidence before authorities act in cases where serious harm may occur. In the environmental context, it was claimed that certainty of harm and evidence of a mechanism of harm to the environment from chemical pollution and the production of oil and gas were not needed for the state to take the action of banning polluting activities under the Swedish Environmental Protection Act of 1969 (Pinto-Bazurco 2020). It was later used to call for action on environmental degradation as articulated in the Rio Declaration of 1992 (UN General Assembly). In its ‘negative’ form, it proposes holding back from taking actions that may cause serious harm even in the face of uncertainty that or how those harms will eventuate, as shown here: ‘Where there are threats of serious or irreversible damage, lack of full scientific certainty shall be not used as a reason for postponing cost-effective measures to prevent environmental degradation’ (UN General Assembly 1992, Principle 15). Since then, PP has been applied in relation to holding back from risky technological advances, and also in a ‘positive form’ where it can be used to propose taking action against (uncertain) serious threats like pandemics and other major disasters (Hartzell-Nichols 2017; Nijsingh et al. 2020; Steel 2014). Whilst there is no single formulation of the principle, most approaches have in common a set of three requirements relating to harm, uncertainty, and authority (Munthe 2020). For the principle to apply, there must be threat of *serious harm,* some *uncertainty* surrounding the likelihood of the threat eventuating and/or its mechanism for coming about, and the existence of an *authority* that can be held responsible for taking precautionary measures against the threat. PP in its positive form plays a vital role in policy-informing ethical analyses of potential threats by shifting the burden of proof: instead of policy action being taken where there is high confidence in harm occurring if action is not taken, it holds that action should be taken unless there is high confidence in the harm *not* occurring. It is therefore more permissive of early action than alternative principles such as cost–benefit analyses would be where the harm is uncertain.

AMR appears to fulfil the requirements for PP to apply (Nijsingh et al. 2020). The threat is serious; there is uncertainty surrounding the likelihood of the harm being so serious due to gaps in our knowledge of the problem (Dadgostar 2019) and surrounding how, exactly, resistance develops and is transmitted across reservoirs. (For instance, evidence of transmission of resistant bacteria between pets and humans is limited in some settings (Jin et al. 2023)).^3^ There are authorities that can be held responsible for taking action on AMR, namely national governments that have agreed to the WHO’s Global Action Plan (GAP) on antimicrobial resistance (2015) and have developed National Action Plans (NAPs) on this basis. The GAP calls on states to take responsibility for contributions to AMR in their jurisdiction, through a series of NAPs that set out goals and strategies for tackling AMR. Many NAPs include plans for precautions against AMR including stewardship measures and monitoring systems to track AMR emergence and spread. Countries are held responsible through NAP evaluation systems systems implemented by the WHO.

If PP applies to AMR, then this suggests that states ought to implement precautionary measures against AMR through their NAPs. Many of these may come in the form of antimicrobial stewardship action, which aims to take care in how antimicrobials are currently used, to optimise usage. For some cases where antibiotics tend to be used more than necessary, this may mean reducing usage; for other cases where there is inadequate access to antibiotics, this may mean increasing access. Measures will need to occur across reservoirs where antimicrobial resistance develops—that means, for instance, intervening in the use of antibiotics to promote growth or health in healthy livestock, use of antifungals alongside fertilisers in food crops, controlling antimicrobial run-off from hospitals and pharmaceutical production facilities, and reducing unnecessary antibiotic use by human patients (Fisher et al. 2022; Larsson and Flach 2022). In many cases, to be effective, the emphasis of precautionary measures will need to be both on changing structures to reduce the need for antibiotics, and on changing behaviours to make people more mindful of the potential resistance-related consequences when using antibiotics. However, many antibiotic stewardship measures, whilst emphasising the need for collective coordination and systemic change, still advocate for stewardship measures that assume individual responsibility for contributions to AMR, and aim at individual behaviour changes (Chandler 2019). Many of these stewardship measures are associated with harms, particularly where they make it much more difficult for already-disadvantaged groups to access antimicrobials, and/or where they are coercive or involve heavy penalties a means of securing broad compliance. To some extent, these potential harms are considered through PP as trade-offs between the harms of not intervening in AMR and the harms imposed on people through a proposed precautionary measure. Formulations of PP that consider this proportionality factor would only recommend those precautionary measures that do not impose disproportionately large harms (Munthe 2020).

## Social justice implications of bans on over-the counter antimicrobials as a precautionary stewardship measure

Both of the cases below are appropriate examples of what PP might be used to justify in terms of stewardship measures, and the latter highlights social justice problems arising from such measures that policymakers relying on PP alone may fail to consider.

First, take a ban on use of certain antibiotics for livestock growth promotion. Regulations in the European Union (EU) have restricted the use in animals of antibiotics that are critical to human health since 1996 (Council of Ministers). The regulations were enforced in Denmark in 1998, and their ruling to ban use of the antibiotic virginiamycin as a growth promoter was confirmed by the EU in 1998 (Council of Ministers 1998). The next year, a challenge was brought to the European Court of First Instance by Pfizer, who argued that there were harms arising from the ban including reductions in animal welfare and increased risks of food contamination. The Court judged that whilst the risks from resistance developing across reservoirs to affect human health were potentially serious, the risks to animal welfare and food safety that Pfizer raised were both preventable and smaller. As a result, it held that: ‘the risk of reducing the effectiveness of human medicinal products […], as a result of cross‐resistance caused by virginiamycin should be avoided’ (European Court of First Instance 2002, paragraph 21). In the end, the policy change in Denmark seems not to have negatively affected farmers or livestock significantly, with farmers reporting an increased cost of 1 Danish krone in real terms in the early 2000s for raising a pig from birth to slaughter, and with pig mortality varying in the early 2000s but stabilising at 1992 rates by 2008 (Levy 2014). Much of the success of such policies is dedicated to effective animal husbandry and farm hygiene measures (Hansen et al. 2008; Levy 2014). The court’s decision in this case has been thoroughly analysed through the lens of the precautionary principle in the literature (Hansen et al. 2008; van Asselt and Vos 2007), and seems itself to incorporate PP as a consideration, with the discussion of serious harm and (uncertain) risk.

By contrast, we might examine a ban on over-the-counter (OTC) sales of antibiotics for human consumption. The Wellcome Trust proposed ‘enhanced gating’ of antibiotics in 2016, including measures to ensure ‘use is routed through healthcare professionals and over-the-counter use is minimised, with due consideration for the need to enhance access to antibiotics in many [low- and middle-income countries] LMICs.’ (Wellcome Trust 2016, p. 11). The WHO’s GAP also discusses OTC sales of antibiotics that ‘contribute to development of antimicrobial resistance’ (World Health Organization 2015, p. 10). The GAP states: ‘Regulation of the use of antimicrobial agents is inadequate or poorly enforced in many areas, such as over-the-counter and Internet sales’ (10). OTC and online antibiotic sales have been shown to occur frequently in some LMICs (Dixon et al. 2021; Nayiga et al. 2020 particularly where healthcare infrastructure (and therefore, prescribers) may be difficult to access or where antibiotics may be needed by day-wage workers who cannot afford to sacrifice income from a day spent at home due to illness (Nabirye et al. 2021). Even in countries where antibiotics are primarily obtained initially by prescription, many courses are not completed, with the remaining antibiotics used ad hoc against later infections. In the European region, up to one in three people use leftover antibiotics or obtained them without a prescription according to a 2022 WHO survey (World Health Organization 2022). Based on the amount of non-prescription antibiotic use described here, banning OTC use may be an effective precautionary measure to reduce the risk of contributions to AMR in the future where multifaceted approaches are taken (Jacobs et al. 2019).^4^ If the harms of enforcing bans are not too high, then their magnitude may be small in comparison to the harm averted from worsening AMR. This measure, if implemented and properly enforced by states through their NAPs, might constitute a precautionary measure that could be justified using PP.

It has already been noted by scholars in the area that PP is a ‘crude and sometimes perverse method’ that can ‘harm instead of help those who are most disadvantaged.’ (Sunstein 2003, p. 37) To assess such claims in the context of stewardship, we might consider both antibiotic bans (livestock and human OTC) through the lens of social justice. According to David Miller’s account, three important aspects of social justice are desert, need, and equality (1999). Using these principles, we can examine questions about the allocation of harms and benefits in a more nuanced way than simple distributive justice allows, according to Miller, due to the contextual factors that the three principles of social justice acknowledge. Consider, first, the EU’s ban on use of antibiotics as growth-promoters. The harms to animal welfare and risk of food contamination were deemed insignificant by the European court, however there was a small harm to farmers in terms of the slightly increased cost of raising livestock. This harm is localised to Danish farmers, and thus unequal across citizens of EU member states. However, the principle of desert may conflict with the principle of equality here, in that we may hold that those who are most accountable for unnecessary contributions to AMR deserve to bear the costs of behaviour change (Johnson and Matlock 2023). With the cost itself being relatively insignificant and farm incomes on the increase for over a decade (Rossi 2019), we may think there are no significant justice concerns raised in this case, and farmers are in no particular need that would justify alterations to our judgement of the social justice of the measure.

Unlike the EU’s livestock antibiotic growth-promoter ban, I argue that banning people’s OTC uses of antibiotics raises clear justice concerns, because of the characteristics of the population that is harmed by eliminating OTC antibiotic use, and the way this indicates underlying social injustice. Again, using Miller’s principle of equality, we might critique the distribution of harms and benefits of this precautionary measure. Those who tend to access antibiotics OTC or online bear the burden of the ban, for the benefit of everyone who would otherwise have suffered from the harms of the ‘silent pandemic’. However, as with the case above, equality might be argued to conflict with desert. It might be claimed that those who use antibiotics without a prescription are at greater risk of contributing to AMR, and thus, deserve to bear the burdens of more stringent stewardship. Indeed, as well as being held accountable, those who access antibiotics OTC may have the greatest capacity to alter the risk of serious harm from AMR, if non-prescription uses of antibiotics are a significant driver of AMR. They may also benefit more from reduced emergence of resistance in communities on a local level. However, the principle of need comes to the fore in this case, as many of those who access antibiotics OTC under current conditions do so out of serious need, as I explain below.

In their examination of OTC antibiotic use in an informal settlement outside Kampala, Nabirye et al. (2021) describe how residents buy antibiotics as they need them and can afford them, on a day-to-day basis, from a local informal drug-store. Residents often work a number of informal jobs, including washing clothes, building, and cooking. They face a number of infectious disease concerns, including cholera and other diarrhoea-related diseases whenever the water that surrounds the settlement on reclaimed land rises. The settlement has poor water and sanitation infrastructure. Residents take antibiotics in order to continue being able to work, with a lost day of work possibly meaning not enough money for medicines, children’s school fees, or food. Antibiotic use, according to Nabirye et al.’s account, is essentially entangled in a web with work and health practices and concerns. The whole is shaped by residents’ limited political power and ability to demand change and shape their environment for better health and work conditions. What would a ban on OTC use mean for a community that so clearly needs access to antibiotics? Assuming penalties were enforced, the residents of settlements like this would face a choice between increased financial hardship from penalties from antibiotic use (where they were still available through illegal sales), and being forced to work whilst ill or stay at home and face further hardships from the inability to work for day-wages. Particularly when some of their primary targets are communities like this, coercive and/or burdensome stewardship measures may essentially be means of ‘punishing the poor’ (Wacquant 2009)—that is, means of shifting the societal gaze onto those living in on the edges of society in informal settlements and their ‘misuses’ of shared resources like effective antibiotics. This has the added effect of shifting the gaze of society away from the structures and systems that have created and perpetuated the conditions that give rise to infection and the need for antibiotic use in these communities. It poses a clear failure of social justice if such policies are justified using PP and implemented. This social justice consideration may render such action unacceptable, or at least unacceptable without tandem measures to counteract the effects of such a ban. It is these potential implications of antimicrobial stewardship measures that have been inadequately addressed in the literature on AMR.

## A possible solution: incorporating justice-related ethical concepts alongside the precautionary principle

I have argued that PP alone, whilst it considers whether the magnitude of harms from the threat vs the precautionary measures(s) proposed is proportionate, does not consider the distribution of harms. I have argued that in ethical analyses of policies to address AMR, where PP is considered alone, this may lead to failures of social justice. This is particularly the case where antimicrobial stewardship measures such as bans on OTC uses of antibiotics that mostly impact disadvantaged populations are implemented as precautionary measures. How should we proceed, given that the need to actually address AMR persists?

The obvious solution is to ensure that ethical analyses of prospective antimicrobial stewardship policies being considered as precautionary measures under the PP include considerations of social justice, through the addition of justice-related ethical concepts or approaches alongside PP, such that PP is never used as a standalone justification for precautionary measures. There are a range of possible ethical justifications for stewardship actions (Johnson 2024a) (some relating particularly well to stewardship measures in agriculture (Johnson 2024b)), and those relating to justice may highlight aspects of global health justice, appropriate distribution of responsibility, or development and sustainability, for example. Whilst ethical analysis itself does not solve social justice problems with antimicrobial stewardship efforts, it does serve to highlight where additions to a policy may need to occur or where alternative measures should be considered.

Issues of social justice are in fact beginning to be considered more in work on AMR. For instance, global justice and development in the context of AMR has been explored already (Reid 2020). In addition, there are a range of justice-related concepts we might borrow from other areas of ethics such as pandemic ethics and research ethics to supplement PP in ethical analysis, including reciprocity (Silva and Smith 2023) benefit-sharing (Simm 2007), and fair collaboration (Parker and Kingori 2016). If we couch efforts against AMR as a matter of global collaboration, or participation in a global trial, then we may better see the need for equitable conversations in designing interventions across global actors, or the need to ensure that the ‘participants’ in the efforts receive benefits in exchange for the burdens they take on through burdensome stewardship.

Alternatively, we might also go further, to ensure that justice is addressed more broadly than the seemingly issue-specific approaches above. Whilst actually deploying a whole account of justice (e.g. David Miller’s social justice (1999)) may be too vague to guide ameliorative action, there are *approaches* to promoting justice in more specific areas, seem to sit between the narrow level of particular concepts, and the breadth of whole accounts. One approach to justice that heavily emphasises issues of social justice but is also effectively used to guide action and create change is that of a ‘just transition’ (McCauley and Heffron 2018). The just transition framing has been used in the climate governance context to highlight many different kinds of injustices that occur when we face global threats that make transition to another way of life necessary (whether that be adaptation to a changed climate, or a post-antibiotic future). In the climate, energy, and environment context, the just transition approach highlights injustices experienced by, for instance, coal miners who are left behind by state efforts to move away from coal as an energy source and toward a green-energy future. It has been used both to highlight problems (in this case, one of leaving behind vulnerable workers and their communities) and potential solutions (for instance, requiring energy providers to commit to climate change adaptation and mitigation measures before licensing) (McCauley and Heffron 2018).

In the AMR space, too, there is work being done to defining and implementing a just transition approach to highlight the risks to populations of transitioning toward a lower-antibiotic-use, antibiotic-sustainable, or even post-antibiotic future (Just Transitions Working Group 2023). The approach as applied here looks to incorporate various other aspects of justice, as well, to address not only social justice, but also concerns that might be raised during a transition in the areas of procedural justice, epistemic justice, and global conceptions such as Ubuntu justice and Confucian justice. This is important given concerns that antimicrobial stewardship efforts may raise that relate not only to burdens on disadvantaged populations, but also, for instance, to the lack of recognition both of traditional knowledge on antibiotic alternatives (Oladunjoye et al. 2022) and of effective stewardship efforts already implemented in LMIC settings (Gebretekle et al. 2018; Lam et al. 2021). In response to proposed precautionary stewardship measures such as bans on OTC antibiotic sales, a just transitions approach can recognise concerns ranging from the social justice points I have already raised, to those regarding how to more equitably involve stakeholders in decision-making concerning stewardship measures and ensure that the voices of people living in communities where antibiotics form a part of essential infrastructure are heard. Approaches like a just transition, if incorporated into ethical analysis and policymaking alongside PP, can better recognise and address injustices that might arise through measures used to transition toward a future where antibiotics are used differently to today.

## Articles in this special issue

The articles in this special issue of *Monash Bioethics Review* all centre around the theme of AMR and social justice. The interpretations of what justice requires vary, as do emphases on the links between social justice and distribution, systems-level change, deliberative processes, epistemic justice, or other ideas. In my view, it is both valid and important that there are different conceptions and emphases present. As newer interpretations of a ‘just transition’ emphasise (Varadan et al. 2023), different justice issues concerning AMR arise in different contexts, and they require different analyses and mechanisms of response.

To start, Lynch and colleagues provide a systematic scoping review for this issue, with a focus on AMR justice issues in relation to gender and intersectional identities (2024). They argue that in the LMIC context, gender plays a significant but under-recognised role in determining AMR risk and vulnerability. As a result, the effects of blanket strategies to address AMR vary in effectiveness and their associated burdens for women, particularly those who also face disadvantages associated with race, class, or ability identities. This justice issue may be particularly important to assess in LMIC contexts due to the increased burden of AMR, and particularly in any LMIC contexts where women bear greater care burdens, have less access to sanitation, or may face greater power differentials in household settings.

In the setting of residential aged care facilities, in their article in this issue, Williams, Chawraingern, and Degeling explore value trade-offs involved in antimicrobial stewardship through a relational lens (2024). This allows them to emphasise both the important role that antibiotics play in this setting as a care function according to adult children of aged care residents. They explore the injustices that may arise where stewardship measures morally burden both family, and facility workers who are primarily concerned with the wellbeing of often-vulnerable residents for with system failures in adequate design of residential aged care facilities.

Sudenkaarne and Butcher incorporate intersectionality in their discussion of justice and AMR, as well (2024). They propose that vulnerability (to the risks of AMR) is contingent on circumstance rather than identity. As a result, it should be divorced from discussions of autonomy, and from anthropocentrism. Using examples such as Bangladeshi export aquaculture, they highlight particularly this latter aspect of the importance of avoiding anthropocentrism. They note links between seafood, climate, bioaccumulation, and human health, and advocate for an approach to justice that supports One Health. This would necessarily include more-than-human ideas of justice. The suggested best contender for a framework is a queer feminist posthuman framework.

Sudenkaarne builds on the paper above in the next work (2024), where she expands on the idea of a queer feminist posthuman framework to assess harms to in the AMR context. She considers harms to non-human sentient animals from antimicrobial use and stewardship measures alike, and the injustice inherent in assigning value only or primarily to human ways of being. The author also examines oppressive structures imposed on women and LGBTQI + people, which create vulnerability, for instance to further control by dominant groups in the name of antimicrobial stewardship.

McKnight et al. complement these transdisciplinary approaches to AMR and social justice with their examination of pregnant people’s experiences of treatment for bacterial infections in pregnancy (2024). They are concerned with reproductive justice, and the question of who is most likely to make sacrifices through foregoing treatment, for the sake of protecting antibiotic effectiveness for future generations. They present the case of a pregnant woman with intersecting identities as a migrant and person of colour, highlighting how the factors that affect how she seeks treatment are various and depend on aspects of identity and situation. This implies that there may be different needs and appropriate approaches to stewardship and treatment options depending on an individual’s intersecting identities and context.

Fumagalli is the only author in this special issue who primarily looks at environmental AMR (2024). In his analysis of pharmaceutical pollution as a driver of AMR, he proposes the leveraging of market approval mechanisms for the production of pharmaceuticals as an effective means of influencing producer behaviours. This is a matter of justice insofar as various specific regulatory options may impact people differently, for instance reducing access to effective medications for those in greatest need for stronger options. What’s more, finding a solution to environmental pollution driving AMR is also a matter of justice when we consider the presence of superbugs in waterways serving communities who live close to pharmaceutical production facilities (Rayan 2023), and may face intersections of economic and other disadvantages.

Finally, Mutua and colleagues focus on antibiotic use in humans and animals in East Africa (2024). They break questions of justice down into three levels: institutional morality, public discourse, and embodied experience. Their analysis highlights discrepancies between our attributions of ‘inappropriate’ behaviours by individuals using and prescribing antibiotics and institutional morality which allows shortcomings in health systems and service delivery to persist. The labels we apply to particular antibiotic behaviours—‘irrational’, ‘misuse’, ‘abuse’, ‘imprudent’—can be harmful in themselves. What’s more, they can perpetuate injustice where they are applied without consideration of how differences in the resourcing and governance of institutional settings and public systems in which individuals using antibiotics ‘inappropriately’ operate affect the reasonable options available to them. These valuable insights provide a final reminder that questions of justice arise at every level and facet of analysis of antimicrobial resistance, whether scientific, linguistic or otherwise.

## Conclusion

AMR poses a threat of serious harm, and this complex problem will require a sustained, multisectoral approach to avert the worst outcomes. Precautionary measures including some burdensome antimicrobial stewardship policies may need to be implemented by countries working together to address AMR. However, PP should not be used as a standalone concept for ethically evaluating precautionary measures. Instead, to identify and address social justice problems that may arise or be exacerbated by certain stewardship efforts such as OTC antibiotic bans, other justice-related concepts such as a just transition approach need to be incorporated into ethical policymaking. With thanks to the contributing authors to this special issue, I hope that the collection enables readers to examine further the measures proposed to curb AMR, and what can be done to better promote social justice through such measures.

## Footnotes

1 Note that I do not deal here with questions about certain harms. There are certain negative consequences of AMR that are already happening, and for which there is good evidence, particularly in settings with effective monitoring and surveillance of resistance in hospitals (National Academies of Sciences, Engineering, and Medicine 2021). Where there is no uncertainty about serious harms, PP is unnecessary, and cost–benefit analyses, application to the harm principle, or a duty of easy rescue may be used as ethical justification for stewardship policy instead. However, there is significant uncertainty remaining concerning some of the more serious harms of AMR, such as deaths to future people and loss of effectiveness of antibiotics of last resort. Therefore, PP is still appropriate, and a useful tool where there is a lack of strong enough evidence for particularly serious harms.

2 I use principle here as a cover-all term for what may be more appropriately characterised as a collection of slightly varying approaches to uncertain yet serious harm, which share an emphasis on the avoidance of serious yet uncertain future harm through precautionary action. Note that PP was initially used to argue *against* taking actions that were deemed possible to result in serious harm, and as such the ‘precautionary measure’ was ceasing the action (such as development of a dangerous new technology).

3 Although, for the view that there is no uncertainty left concerning the serious harms AMR will pose, see: Abimbola et al. (2021).

4 Although, not all bans on OTC antibiotic use prove effective (Rodrigues et al. 2021).
